# The complete mitochondrial genome of a skipper *Burara striata* (Lepidoptera: Hesperiidae)

**DOI:** 10.1080/23802359.2017.1298416

**Published:** 2017-03-10

**Authors:** Jing Zhang, Qian Cong, Jinhui Shen, Rongjiang Wang, Nick V. Grishin

**Affiliations:** a Department of Biophysics and Biochemistry, University of Texas Southwestern Medical Center, Dallas, TX, USA;; b College of Life Sciences, Peking University, Beijing, China;; c Howard Hughes Medical Institute, University of Texas Southwestern Medical Center, Dallas, TX, USA

**Keywords:** Next-generation sequencing, phylogeny, China, Fluted Awlet, Bibasis

## Abstract

We assembled a complete mitogenome of an Asian skipper butterfly *Burara striata* (Hesperiidae, Coeliadinae), the first representative of the genus *Burara*, from next generation sequencing reads. The 15327 bp mitogenome covers 13 protein-coding genes (PCGs), 22 transfer RNA genes (tRNAs), 2 ribosomal RNA genes (rRNAs), and an A + T rich region. Its gene order is typical for mitogenomes of Lepidoptera. Phylogenetic analysis places *Burara striata* as a sister to *Hasora*, and *Choaspes* as a sister to both of these genera.

The Fluted Awlet (*Burara striata*) is a Skipper butterfly (family Hesperiidae) from the subfamily Coeliadinae. This Old World subfamily is characterized by long and thin cylindrical (awl-shaped) third segment of labial palps (Chiba 2009). *Burara striata* is a large butterfly with forewing length reaching 3 centimeters. It is brown above and mostly green below with dark lines along and between the veins giving it a fluted appearance. It is widely distributed in China and Korea (Chiba 2009). Formerly, this skipper and its relatives were placed in the genus *Bibasis*, i.e., *Burara* was considered to be a synonym of *Bibasis* (Evans 1949). However, in many recent works the two genera are being treated as distinct (Vane-Wright & de Jong 2003; Warren et al. 2008; Chiba 2009; Warren et al. 2009). Not only they differ morphologically in genitalic and wing characters, i.e., *Bibasis* possesses typically broader wings with a pale central band or its trace below that is lacking in *Burara* (Chiba 2009), but also in behavior. *Burara* species are crepuscular, but *Bibasis* species are mostly diurnal (Maruyama 1991). For Coeliadinae, complete mitochondrial genomes are currently available only for *Choaspes benjaminii* (Kim et al. 2014) and two species of *Hasora* (Cao et al. 2016; Wang et al. 2016) leaving other genera to be explored.

To better understand the phylogeny of Hesperiidae, we sequenced, assembled and annotated the complete mitogenome of *Burara striata* from the female voucher NVG-5270 collected in China: Sichuan Province, Pingwu County, the Old Creek Nature reserve on 11-Aug-2015. The body of the specimen was preserved in RNAlater and wings are illustrated in Figure 1(a). Methods for genomic DNA extraction, library construction, next-generation sequencing, and computational procedures have been reported by us previously (Shen et al. 2015; Cong & Grishin 2016; Cong et al. 2016a, 2016b; Shen et al. 2016). The mitogenome of *Choaspes benjaminii* (Kim et al. 2014) was used as a reference to search for (“bait”) similar sequence reads using BWA (Li & Durbin 2009). Nearly 1.6% (684400 out of 43857786) of *B. striata* total genomic reads were extracted by BWA for mitogenome assembly (Hahn et al. 2013). The complete mitogenome of *B. striata* was assembled de novo using Platanus (Kajitani et al. 2014) followed by a manual gap-closing procedure.

**Figure 1.**
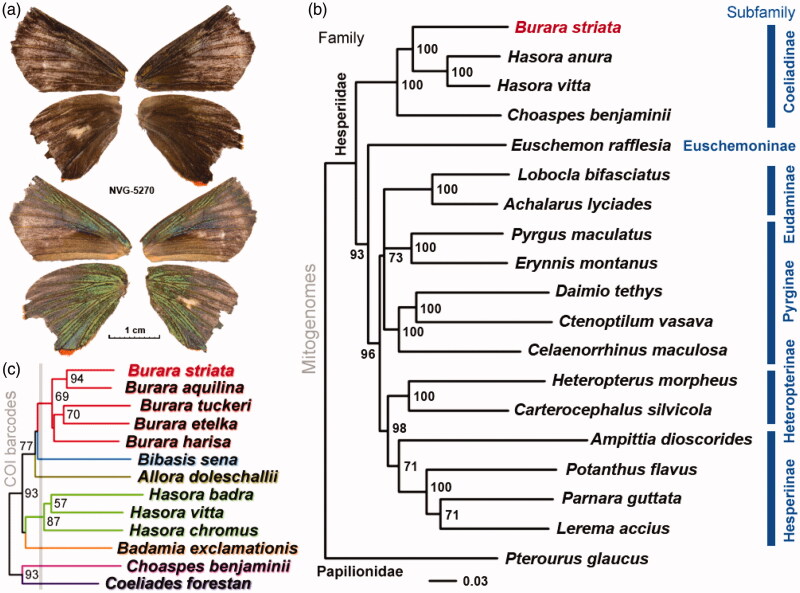
The specimen and trees. (a) *Burara striata* voucher NVG-5270 with mitogenome reported here, dorsal and ventral sides above and below, respectively. (b) Maximum likelihood tree of complete mitogenomes of 18 Hesperiidae species rooted with *Pterourus glaucus* (Papilionidae). Numbers by the nodes show bootstrap support values and branches with bootstrap less than 70% are collapsed. GenBank accessions for sequences are: *Achalarus lyciades* NC_030602.1; *Ampittia dioscorides* KM102732.1; *Burara striata* KY524446; *Celaenorrhinus maculosa* NC_022853.1; *Daimio tethys* NC_024648.1; *Euschemon rafflesia* KY513288; *Erynnis montanus* NC_021427.1; *Hasora anura* NC_027263.1; *Hasora vitta* NC_027170.1; *Heteropterus morpheus* NC_028506.1; *Choaspes benjaminii* NC_024647.1; *Lerema accius* NC_029826.1; *Lobocla bifasciatus* NC_024649.1; *Carterocephalus silvicola* NC_024646.1; *Potanthus flavus* NC_024650.1; *Parnara guttata* NC_029136.1; *Pyrgus maculatus* NC_030192.1; *Ctenoptilum vasava* NC_016704.1; *Papilio glaucus* NC_027252. (c) P-distance BioNJ (Dereeper et al. 2008) dendrogram of COI barcodes of representative Coeliadinae. Identification of these specimens was not checked and may be erroneous for some. Bootstrap values above 50% are shown. Different genera are colored in different colors. Gray vertical line represents a boundary to define the genera. Accessions for sequences are: *Allora doleschallii* KF388929; *Badamia exclamationis* KF391242; *Bibasis sena* KY019679; *Burara aquilina* GU372597; *Burara etelka* JF852007; *Burara harisa* JF852009; *Burara striata* KY524446; *Burara tuckeri* YB-KHC8641; *Choaspes benjaminii* HQ566976; *Coeliades forestan* KY019713; *Hasora badra* JF852078; *Hasora chromus* KF388562; *Hasora vitta* JF852080.

The complete mitogenome of *B. striata* is 15327 bp in length (Genbank: KY524446) and is AT rich, with a base composition of 39.2% A, 40.9% T, 7.6% G, and 12.3% C. It retains the typical insect mitogenome gene set, including 13 PCGs (ND1-6, COX1-3, ND4L, ATP8, ATP6, and CYTB), 22 tRNA genes (two for serine and leucine and one for each of the rest amino acids), 2 ribosomal RNAs (rrnL and rrnS), and an A + T rich D-loop control region. As in many Lepidoptera mitogenomes, the exact start of COX1 gene is unknown, but is probably the codon TTG (Kim et al. 2009). The typical start codon ATN is used in other genes except ND1 which may use GTG as start codon. COX1, COX2, ND4 and ND5 genes have an incomplete stop codon T, and a complete TAA codon is likely formed during mRNA maturation (Ojala et al. 1981; Boore 1999). The length of tRNAs ranges from 62 to 71 bp. The size of the two rRNAs are 1367 and 778 bp, respectively. A 313 bp A + T rich region connects rrnS and tRNA-Met.

To phylogenetically place *Burara striata* within Hesperiidae with available mitogenomes (Hao et al. 2012; Wang et al. 2013; Kim et al. 2014; Wang et al. 2014; Shao et al. 2015; Shen et al. 2015; Cao et al. 2016; Cong & Grishin 2016; Shen et al. 2016; Wang et al. 2016; Zhang et al. 2017), we constructed RaxML (Stamatakis 2006) maximum likelihood tree rooted with *Pterourus glaucus* (Papilionidae) mitogenome (Shen et al. 2015) (Figure 1(b)). The resulting tree topology is consistent with previous phylogenetic studies (Warren et al. 2008, 2009; Sahoo et al. 2016; Zhang et al. 2017): Coeliadinae are the sister to all other Hesperiidae; *Euschemon* is a sister to the rest of Hesperiidae except Coeliadinae; relationship between Eudaminae and Pyrginae is unresolved; and Heteropterinae are the sister to Hesperiinae. Among Coeliadinae with complete mitogenomes, *Burara* is the sister to *Hasora* and *Choaspes* is the sister to them. This topology is consistent with morphology (Chiba 2009) and the most recent DNA study (Sahoo et al. 2016). To further investigate the relationship between Coeliadinae genera and genetic distinctness between *Bibasis* and *Burara*, we constructed a distance dendrogram from representative COI barcodes (Figure 1(c)). *Bibasis* appears to be more separated from the cluster of *Burara* species and is more divergent from them than the species of *Hasora* from each other, but is at about the same distance from *Burara* as *Allora*, which has been considered a good genus. Therefore, it is reasonable to treat *Burara* as a genus different from *Bibasis*.
